# Improved nutrient intake following implementation of the consensus standardised parenteral nutrition formulations in preterm neonates – a before-after intervention study

**DOI:** 10.1186/s12887-014-0309-0

**Published:** 2014-12-17

**Authors:** Srinivas Bolisetty, Pramod Pharande, Lakshman Nirthanakumaran, Timothy Quy-Phong Do, David Osborn, John Smyth, John Sinn, Kei Lui

**Affiliations:** Division of Newborn Services, Royal Hospital for Women, Sydney, Australia; University of New South Wales, Sydney, Australia; University of Sydney and RPA Newborn Care, Royal Prince Alfred Hospital, Sydney, Australia; Department of Neonatology, Royal North Shore Hospital, University of Sydney and, Sydney, Australia; Division of Newborn Services, Royal Hospital for Women, Barker Street, Locked Bag 2000, Randwick, 2031 NSW Australia

**Keywords:** Parenteral nutrition, Newborn, Standardised formulation

## Abstract

**Background:**

New standardised parenteral nutrition (SPN) formulations were implemented in July 2011 in many neonatal intensive care units in New South Wales following consensus group recommendations. The aim was to evaluate the efficacy and safety profile of new consensus formulations in preterm infants born less than 32 weeks.

**Methods:**

A before-after intervention study conducted at a tertiary neonatal intensive care unit. Data from the post-consensus cohort (2011 to 2012) were prospectively collected and compared retrospectively with a pre-consensus cohort of neonates (2010).

**Results:**

Post-consensus group commenced parenteral nutrition (PN) significantly earlier (6 v 11 hours of age, p 0.005). In comparison to the pre-consensus cohort, there was a higher protein intake from day 1 (1.34 v 0.49 g/kg, p 0.000) to day 7 (3.55 v 2.35 g/kg, p 0.000), higher caloric intake from day 1 (30 v 26 kcal/kg, p 0.004) to day 3 (64 v 62 kcal/kg, p 0.026), and less daily fluid intake from day 3 (105.8 v 113.8 mL/kg, p 0.011) to day 7 (148.8 v 156.2 mL/kg, p 0.025), and reduced duration of lipid therapy (253 v 475 hr, p 0.011). This group also had a significantly greater weight gain in the first 4 weeks (285 v 220 g, p 0.003).

**Conclusions:**

New consensus SPN solutions provided better protein intake in the first 7 days and were associated with greater weight gain in the first 4 weeks. However, protein intake on day 1 was below the consensus goal of 2 g/kg/day.

**Electronic supplementary material:**

The online version of this article (doi:10.1186/s12887-014-0309-0) contains supplementary material, which is available to authorized users.

## Background

Parenteral nutrition (PN) is an essential component in the management of many newborn infants, particularly premature low birth weight infants admitted to Newborn Intensive Care Units (NICUs) [1]. In many NICUs in Australia and New Zealand (ANZ), PN is provided by standardised stock solutions rather than individualised solutions prescribed and prepared for each infant. Standardized PN (SPN) solutions have been shown to provide improved nutrition to infants compared to individualized PN solutions [2]. Until recently, each NICU in ANZ used their own standardised PN solutions. In 2010, a multidisciplinary group was formed to achieve a consensus on the formulations acceptable to the majority of the NICUs. Literature review was undertaken for each nutrient and recommendations were developed in a series of meetings held between November 2010 and April 2011. Three standard and 2 optional amino acid/dextrose formulations and one lipid emulsion were in the consensus. The detailed outcomes and recommendations of the consensus group have been published [3].

Royal Hospital for Women (RHW) is a tertiary perinatal centre in New South Wales with over 4000 deliveries per year. Neonatal Intensive Care Unit (NICU) at RHW provides the services for newborns with complex medical and surgical conditions. RHW was among the first 3 NICUs in NSW that implemented the new management protocol from July 2011.

The main objective of this study was to evaluate the nutritional intakes and weight gain in preterm infants born less than 32 weeks managed in our NICU using the new consensus SPN management protocol.

We aimed to study the following: (1) determine daily fluid, essential nutrient (protein, carbohydrate, lipids) and energy intakes received through parenteral and enteral nutrition in the first week and on day 14, 21 and 28 if the infant was still in NICU; (2) identify the incidence of electrolyte and other metabolic disturbances in the first week; (3) examine the limiting factors in achieving projected nutritional intake from the consensus PN solutions; and (4) compare the PN and enteral nutritional intakes and growth patterns between two cohort groups.

We hypothesised that protein and energy intakes of infants would improve with implementation of new consensus SPN formulations in 2011.

### Methods

This is a before-after intervention study involving 2 cohorts of preterm infants born less than 32 weeks. The post-consensus cohort included infants admitted to RHW NICU between 1^st^ August 2011 and 31^st^ July 2012. All data from this cohort were prospectively collected. A pre-consensus cohort acted as control and included infants admitted between 1^st^ January 2010 and 31^st^ December 2010. Data from this cohort were collected retrospectively. There was a 6 month transition period between 2 cohorts during which the new consensus PN management protocol was progressively introduced with regular education and training of staff with full implementation in July 2011. We excluded neonates with major congenital malformations and chromosomal anomalies and those who were born elsewhere and transferred to RHW after 24 hours of age.

Primary outcome measures were fluid, energy and major nutrient intakes during the first week of life, days 14, 21 and 28. Secondary outcomes measures were biochemical parameters including daily pH, PCO2, HCO3, base excess, plasma ionized calcium, plasma sodium, chloride, urea, creatinine, albumin and magnesium for the first 7 days of life.

Liver function tests, calcium, phosphate and magnesium were done weekly in the first 4 weeks of life and then fortnightly to monthly until 36 weeks corrected age or discharge. Weight percentiles were based on the Australian birth weight percentiles by gestational age [4].

Statistical analyses were performed using SPSS version 20.0. Data are presented as number (%) or median (Interquartile range, IQR). The clinical and demographic characteristics of the infants were compared using chi-square test with continuity correction, t-test, and Mann–Whitney U-test where appropriate. All p values were two-sided and the p < 0.05 was considered statistically significant.

The study was approved by the South Eastern Sydney and Illawarra Area Health Service Human Research Ethics Committee-Northern Sector.

PN formulations used in 2010 (pre consensus cohort) and the new consensus PN formulations introduced in 2011 (Post consensus cohort) are reported in Additional files 1 and 2 respectively. The major difference in the formulations (Additional file 3) is the protein content. Using 2010 solutions the infant received a maximum 3 g/kg/day of protein at 150 ml/kg/day, whilst in 2011 the infant received a maximum 4 g/kg/day of protein at 135 ml/kg/day. Since 2011 the water content of lipid emulsions (15 ml/kg at 3 g/kg/day) has been included in the total fluid intake. There were also several changes to sodium, chloride, acetate, calcium, magnesium, trace elements and heparin in the PN formulations.

## Results

**Figure 1:**
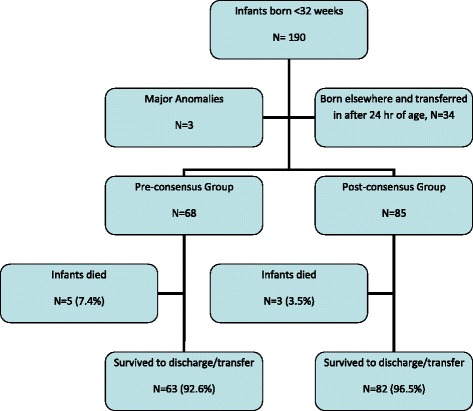
**Study population.**

Figure 1 shows the study population. Between January 1^st^ 2010 - December 31^st^ 2010 and August 1^st^ 2011-July 31^st^ 2012, a total of 190 neonates born with gestational age <32 weeks were admitted. Three neonates with major congenital anomalies (tracheo-esophageal fistula, meconium ileus with cystic fibrosis and trisomy 9) and 34 neonates who were born elsewhere and transferred to our NICU after 24 hours of age were excluded. The remaining 153 neonates who met eligibility criteria were included in the study and divided into pre (N = 68) and post-consensus (N = 85) groups.

The maternal and neonatal characteristics at birth were similar in both groups (Table 1).

**Table 1:** **Perinatal and neonatal characteristics of the study population**

	**Pre-consensus PN Group (n = 68)**	**Post-consensus PN Group (n = 85)**	**P value**
Gestational age at birth, weeks (Median ± IQR)	28.5 (3)	29 (2)	0.218
Birth weight, g (Median ± IQR)	1110 (580)	1240 (614)	0.243
BW percentile, (Median ± IQR)	47.5 (44)	45 (41)	0.868
SGA, <10^th^ percentile	5 (7.4%)	14 (16.5)	0.089
Male gender	34 (50%)	40 (47.1%)	0.718
Antenatal steroids	58 (85.3%)	70 (82.3%)	0.352
Chorioamnionitis	11 (16.2%)	8 (9.4%)	0.207
Pre eclampsia	14 (20.6%)	22 (25.9%)	0.443
Outborn	3 (4.4%)	5 (5.9%)	0.685
SVD	18 (26.5%)	31 (36.5%)	0.188
Apgar <7 at 5 min	12 (17.6%)	11 (12.9%)	0.418

Numbers (%) are given unless indicated.

Daily nutritional intakes for the first week and on days 14, 21 and 28 were measured and a summary is reported in Table 2. Some infants were transferred to non-tertiary care units for ongoing care and nutrient data were available only for their stay in our NICU.

**Table 2:** **Nutritional intakes of the study population**

	**Pre-consensus PN Group (n = 68)**	**Post-consensus PN Group (n = 85)**	**P Value**
Age at commencement,			
hr			
AA	11 (25)	6 (10)	0.005
Lipids	19 (29)	24.5 (26)	0.394
Duration of TPN, hr			
AA	483 (357)	301.5 (205)	0.128
Lipids	475 (357)	253.5 (180)	0.011
Age at 1 g/kg/day of lipid, hr	19 (29)	26 (30)	0.294
Age at 2 g/kg/day of lipid, hr	46 (22)	56.50 (28)	0.004
Age at 3 g/kg/day of lipid, hr	73 (24)	82 (40)	0.010
**Day 1**			
Protein, g/kg	0.49 (0.83)	1.34 (1.16)	0.000
Lipid, g/kg	0.17 (0.73)	0.09 (0.6)	0.295
Calories, kcal/kg	26.49 (6.86)	30.23 (11.04)	0.004
Total fluid (ml/kg)	63.15 (14)	62.24 (16.3)	0.572
AA/Dex (ml/kg)	8.59 (39.6)	38.76 (37.92)	
**Day 3**			
Protein, g/kg	1.73 (0.45)	2.86 (0.81)	0.000
Lipid, g/kg	1.98 (0.77)	1.80 (1.15)	0.190
Calories, kcal/kg	62.20 (7.41)	64.24 (14.61)	0.026
Total fluid (ml/kg)	113.76 (19.07)	105.88 (15.07)	0.011
AA/Dex (ml/kg)	84.42 (25)	84.40 (27.07)	
**Day 7**			
Protein, g/kg	2.35 (0.78)	3.55 (0.89)	0.000
Lipid, g/kg	3.24 (1.77)	3.44 (1.55)	0.784
Calories, kcal/kg	90.2 (26.56)	95.92 (14.28)	0.134
Total fluid (ml/kg)	156.20 (27.3)	148.88 (11.6)	0.025
AA/Dex (ml/kg)	91.26 (47.07)	88.60 (70.19)	
**Day 14**			
Protein, g/kg	2.58 (0.73)	3.58 (1.67)	0.066
Lipid, g/kg	5.1 (2.8)	5.98 (2.81)	0.121
Calories, kcal/kg	101.94 (23.36)	106.3 (36.97)	0.515
Total fluid (ml/kg)	155.74 (18.5)	155.34 (24.9)	0.580
**Day 21**			
Protein, g/kg	2.87 (1.36)	3.70 (0.59)	0.005
Lipid, g/kg	6.06 (2.48)	6.45 (1.57)	0.127
Calories, kcal/kg	113.65 (31.88)	122.66 (33.23)	0.139
Total fluid (ml/kg)	155.39 (18)	161.19 (21.7)	0.34
**Day 28**			
Protein, g/kg	3.42 (1.48)	3.69 (0.46)	0.002
Lipid, g/kg	6.16 (1.47)	6.62 (0.82)	0.010
Calories, kcal/kg	117 (27.02)	126.8 (22.66)	0.010
Total fluid (ml/kg)	155.93 (17.8)	162.47 (17.5)	0.034

All numbers are Median ± IQR.

Age of commencement of amino acid (AA) was significantly earlier in the post-consensus group compared to the pre-consensus group (6 hours v 11 hours of age, p 0.005), but the duration of AA supplementation remained similar. Median AA intake was significantly higher from day 1 (1.34 g/kg) to day 7 (3.55 g/kg) in the post-consensus group and continued to be higher on days 21 and 28 though the majority of neonates were on enteral feeds by that time. Age of commencement of lipid was similar in both groups (29 hours v 26 hours of age) but the duration was significantly reduced in the post-consensus group (253 hours v 475 hr, p 0.011). Daily caloric intake was significantly higher from day 1 to day 3 (30, 48 and 64 kcal/kg respectively) in the post-consensus group as compared to the pre-consensus group (26, 44, 62 kcal/kg respectively). However, calorie intakes were similar between the 2 groups subsequently. Daily fluid intake remained similar in the first 2 days. From day 3 to day 7, the post-consensus group received significantly less daily fluid intake in comparison to other group.

Biochemical parameters monitored during the study period are shown in Table 3. Arterial/capillary pH remained similar in both cohorts from day 1 to day 3. From day 4 to day 7, infants in the post-consensus group had higher pH (>7.3) along with significantly higher bicarbonate (26 v 22 mmol/L) and positive base excess (1.7 v −2.6 mmol/L). This effect disappeared on days 14, 21 and 28 as pH, bicarbonate and base excess values remained similar between the 2 groups. During the study period arterial/capillary pCO_2_ remained similar in both groups.

Urea was significantly higher from day 1 (5.6 v 4.1 mmol/L, p 0.012) and increased slowly up to day 7 (8.8 v 4.8 mmol/L, p 0.000) in the post-consensus group. None of the neonates from either study group had cholestasis.

Clinical outcomes are shown in Table 4. The post-consensus PN group had significantly less days of respiratory support (20.2 days) compared to the pre- consensus PN group (20.2 days v 34 days, p 0.009). Rates of chronic lung disease trended lower in the post-consensus group but did not reach statistical significance (p 0.056). Discharge weight percentiles trended higher in the post-consensus group but did not reach statistical significance. Other neonatal mordities were similar between the 2 groups.

**Table 3:** **Biochemical parameters in the first 7 days of life**

	**Pre-consensus PN Group (n = 68)**	**Post-consensus PN Group (n = 85)**	**P Value**
**Day 1**			
pH	7.30 (0.3)	7.30 (0.1)	0.112
PCO2	43 (18.8)	48 (15)	0.244
HCO3	22.65 (4.8)	23.7 (4)	0.073
Base excess	−2.3 (4.3)	−1.55 (4.3)	0.091
Sodium	138 (4)	139 (3)	0.136
Chloride	108.5 (5)	107 (6)	0.269
Urea	4.15 (2.3)	5.60 (2.4)	0.012
Creatinine	66.5 (24)	68 (19)	0.940
**Day 3**			
pH	7.23 (0.3)	7.31 (0.1)	0.09
PCO2	40 (11.8)	42 (12)	0.998
HCO3	19.8 (3.4)	20.65 (4.1)	0.564
Base excess	−4.8 (4.4)	−4.65 (5.2)	0.684
Sodium	141.5 (4)	144 (6)	0.000
Chloride	112 (5)	111 (6)	0.144
Urea	6.15 (3.5)	8.20 (5.0)	0.000
Creatinine	78.5 (27)	67 (26)	0.006
**Day 7**			
pH	7.29 (0.12)	7.37 (0.07)	0.000
PCO2	45.65 (11)	46.5 (9.8)	0.483
HCO3	22.5 (3.48)	26.15 (4.65)	0.000
Base excess	−2.6 (3.73)	1.75 (4.13)	0.000
Sodium	136 (4)	139 (5)	0.001
Chloride	106.5 (6)	103 (4)	0.000
Urea	4.4 (3.3)	7.7 (3.9)	0.000
Creatinine	61 (33)	57 (20)	0.378

All values are mmol/L except pH.

**Table 4:** **Neonatal outcomes**

	**Pre-consensus**	**Post-consensus PN Group**	**P Value**
	**PN Group (N = 68)**	**(N = 85)**	
Hypotension needing inotropic support	7 (10.3)	6 (7.1) (OR 0.66, 95% CI 0.21, 2.07)	0.476
Days of respiratory support (Mean ± SD)	34 (±35.90)	20.2 (±25.72)	0.009
CLD	19 (27.9)	13 (15.3) (OR 0.46, 95% CI 0.21, 1.02)	0.056
Sepsis (Early and late onset)	14 (20.6)	10 (11.7) (OR 0.51, 95% CI 0.21, 1.24)	0.315
PDA needing treatment	24 (35)	20 (23.5) (OR 0.56, 95% CI 0.27, 1.14)	0.277
NEC > stage II	6 (8.8)	3 (3.5) (OR 0.37, 95% CI 0.09, 1.57)	0.167
ROP stage III and above	4 (5.9)	3 (3.6) (OR 0.58, 95% CI 0.12, 2.70)	0.510
IVH > Grade II	7 (10.3)	7 (8.3) (OR 0.78, 95% CI 0.26, 2.34)	0.678
PMA at discharge/Transfer, wk	36.7 (±5.01)	34.8 (±3.76)	0.013
Discharge weight, g (Mean ± SD)	2141 (±796.24)	1978 (±672.74)	0.183
Discharge weight percentile (Mean ± SD)	12.6 (±15.28)	17.2 (±16.9)	0.016
Weight gain by 4 weeks age, g (Mean ± SD)	218.8 (±155.47)	264.9 (±234.15)	0.003
Mortality	5 (7.35)	3 (3.52) (OR 0.46, 95% CI 0.10, 2.00)	0.291

Numbers (%) are given unless indicated. PMA, Postmenstrual age in weeks.

## Discussion

The detailed consensus agreement of the neonatal PN consensus group was published previously. Main points of agreement were to (1) provide a protein intake of 2 g/kg/day on day 1 and to increase the maximum to 4 g/kg/day by day 5; (2) restricted fluid regimen with 60 ml/kg/day on day 1 to a maximum parenteral fluid intake of 150 ml/kg/day; (3) inclusion of lipid emulsion in the total parenteral fluid intake; and (4) partial replacement of chloride with acetate to reduce hyperchloremic metabolic acidosis.

Our results show that the post-consensus group received significantly higher parenteral protein and lower fluid intake in the first few days in comparison to the pre-consensus group. Higher protein intakes coincided with higher blood urea nitrogen levels in the post-consensus group. Consensus PN solutions were designed to provide 2 g/kg/day of amino acid on day 1 and to increase to 4 g/kg/day maximum [5-8]. However, the average starting protein intake achieved in our cohort was 1.34 g/kg/day which was below the goal of 2 g/kg/day on day 1. Although neonates received 62 ml/kg/day of intravenous fluids on day 1, the amount of amino acid/dextrose solution received was only 39 ml/kg/day. A PN solution with 5% amino acids would be required to provide 2 g/kg/day of protein at 40 ml/kg/day. Our consensus starter solution contained 3.3% amino acids, the maximum amount of amino acids for which physicochemical stability was guaranteed by the pharmaceutical company during the consensus meetings.

There is insufficient evidence to determine optimal timing of introduction of lipid. Systematic review of trials of early introduction of lipid found no significant difference in outcomes comparing early versus late introduction [9]. Consensus was that lipids can be started with the introduction of AAD solutions [10]. There was no consensus among the consensus group on time of initiation of lipid in infants <800 g. ESPGHAN 2005 recommends lipid emulsion should be started no later than on the third day in any neonate who is not sufficiently enterally fed [1]. In this study lipid emulsion infusion was started on day 1 along with AAD solutions in all gestation age groups. During the pre-consensus period, triglyceride levels were not monitored and lipid was increased by 1 g/kg each day to maximum of 3 g/kg/day. In the post-consensus group triglyceride levels were monitored before increasing the lipid dose. Plasma triglycerides were measured before each increase to 3 g/kg/day and then 48 hr later and then weekly thereafter as long as the infant was on lipid emulsions. If triglyceride levels were >2.8 mmol/L, lipid emulsions were reduced by 1 g/kg/day but continued at least at 0.5 g/kg/day to prevent essential fatty acid deficiency [1]. Lipid intakes were not significantly different between the 2 groups. Rate of increase in lipid emulsion was significantly slower in the post-consensus group. In the post-consensus group, lipid infusion duration was significantly less (10.5 v 19.7 days). This corresponds with our new guidelines of ceasing lipid emulsions once the enteral milk volume reaches 100 ml/kg/day which provides an enteral lipid intake of 3.5 g/kg/day.

There were no major electrolyte disturbances (hyponatremia, hypernatremia, hyperkalemia or hypokalemia) in either study group. Though the median duration of PN solutions was 20.1 days (pre-consensus) and 12.5 days (post-consensus), none of the infants developed cholestasis. The reasons could be multifactorial. Our PN solutions do not contain copper and manganese trace elements which may be associated with cholestatsis [1]. None of the study infants were diagnosed with metabolic bone disease and calcium, phosphate and alkaline phosphate levels were within normal limits.

The post-consensus group had a significantly greater weight gain in the first 4 weeks compared to the pre-consesus group. However, there was no significant difference in weight in the post-consensus group at transfer/discharge likely to reflect subsequent enteral intakes and which is consistent with the study by Clarke et al. [6]. There was a trend towards higher discharge weight percentiles in the later cohort. Duration of respiratory support was signficantly lower in the post-consensus group although the difference in incidence of chronic lung disease did not reach statistical significance. It is possible that the reduced duration of respiratory support in the post-consensus group could be related to the restricted fluid intake and/or monitoring for lipid intolerance and also the simultaneous introduction of a “Golden-hour” protocol targeting the immediate management of the very preterm infant at birth to reduce chronic lung disease.

Hyperchloremic metabolic acidosis is a common problem in very low birth weight infants [11]. In our NICU, we have been using parenteral nutrition solutions that partially replace chloride with acetate for some years. New consensus SPN formulations contain more acetate in comparison to pre-consensus solutions. The post-consensus group had a higher pH, higher bicarbonate and normal chloride levels between day 4 and 7. These results are consistent with the acetate supplementation study in neonates [11]. One of the side effects of acetate supplementation is a higher PCO_2_. However, PCO_2_ levels were similar between the 2 groups in our study.

The purpose of providing parenteral and enteral nutrition in preterm infants is to not only achieve the intrauterine-like growth rates but also improve the mortality, morbidities and long term neurodevelopmental outcomes. Early “aggressive” parenteral nutrition is now the recommended practice for very low birthweight infants [1,12,13]. The current practice in many NICUs in Australia is to use standard pre-mixed formulations. Our group developed consensus guidelines based on both the evidence and the availability, compatibility and the ease of implementation of the formulations across the region in a safe and effective way. Our philosophy was that the provision of parenteral nutrition cannot be seen in isolation but in the context of the other interventions such as the amount of fluids given to these infants. However our formulations were designed in such a way that infants receive protein, lipid and energy intakes of 2 g/kg/day, 1 g/kg/day and 40 kcal/kg/day (Starter PN, Annexure 2) on day 1 of life. In an effort to do this, our starter PN formulation contains 33 g/L of amino acids (Primene 10%) and 100 g/L of glucose. This formulation is lot more concentrated than the formulations used in some of the recent observational studies published [16]. Herrmann and collegues demonstrated a better postnatal growth with over 50% of infants <30 weeks gestation remained above the 10^th^ percentile of intrauterine growth by providing early amino acids and energy intakes of at least 50 kcal/kg/day after the first 24 hours of life in 2003–2007 cohort of 84 infants [14]. They increased the calories to 50–70 kcal/kg/day beginning 1 hour after birth in a subsequent 2009–2010 cohort involving 54 infants [15]. There was a significant increase in the amount of fluids in the first 2 days of life compared to 2003–2007 cohort. While weight changes were similar in the first few days between the 2 cohorts, there was no improvement in 10^th^ percentile growth at 36 weeks postmenstrual age compared to 2003–2007 cohort. There was also a significant increase in the incidence of medical treatment for PDA (58% v 25%), insulin for hyperglycaemia (26% v 12%) and conjugated bilirubin >34 μmol/L (36% v 20%). There was also a trend toward increased incidence of NEC (8% v 1%, p 0.08). While the lack of weight improvement at 36 weeks can be explained by changes in enteral nutrition practice, some of the morbidities may be explained by increased fluid intake [16]. Senterre and colleagues from Belgium studied 102 infants <1250 g at birth [17]. They provided mean intakes of 38 kcal/kg/day of energy and 2.4 g/kg/day of protein on day 1 followed by mean intakes of 80 kcal/kg/day and 3.2 g/kg/day of protein in the first week. On average from birth to discharge, 122 kcal/kg/day and 3.7 g/kg/day of protein were administered. They limited the postnatal weight loss to the first 3 days of life, and birthweight was regained after 7 days. Their nutrition and fluid protocol in the first few days of life was somewhat similar to ours. However, the strength in Senterre’s policy was not only to optimize PN but also enteral nutrition by ensuring optimal enteral protein intake. It is also interesting to note their policy of discontinuing PN if enteral feeds are well tolerated once 120 mL/kg/day have been achieved and tolerated. We introduced a similar policy in our consensus. This explains the reason why the duration of PN in the post-consensus cohort was less than the pre-consesus cohort.

We acknowledge the limitations in this study. Infants did not receive the intended protein and energy intakes in the first few days life. On the first day of life, aminoacid/dextrose solution was commenced around 6 hours of life, which was earlier than the pre-consensus group but not from birth. The 2011–2012 post-consensus group for this study was immediately after the introduction of the consensus guidelines. There was a 6-month transition period (January 2011-June 2011) during which staff was given education, training and understaning on the importance of early nutrition and the need for change in policy in the NICU. There were 2 incidents in our NICU during this transition phase with lipaemic blood and very high plasma triglycerides. This resulted in a conservative approach to the commencement of lipids and strict monitoring of lipids. There was also a concern in the NICU that the incidence of chronic lung disease was high and there was a quality improvement project around the same time monitoring the fluid intake to reduce the excess fluid intake. All these factors might have impacted on the nutrient intakes received by the infants during the study period. After the completion of the enrolment for this study in July 2012, we tightened the policy and aimed to commence the PN solutions includng the lipids within 2 hours of life. We hope to analyse the outcomes for this group soon. Other limitation in our study was the lack of complete enteral and parenteral intake data from birth to discharge to determine any improvements or variation between the cohorts.

## Conclusion

In summary, consensus PN solutions provided higher protein intake in the first few days of life and were associated with higher weight gain in the first 4 weeks despite restricted fluid intake in comparison to the pre-consensus group. However, protein intake in the first 2 days can be further improved by increasing the amino acid content in the formulation provided physico-chemical stability of such formulations is assured.

## Additional files

Additional file 1:
**Standardised Amino acid-Dextrose Formulations from January 2010 to June 2011 (Pre-consensus cohort).** The table describes the composition of standardised PN formulations in the pre-consensus cohort. Additional file 2:
**Standardised Amino acid-Dextrose formulations from July 2011 (post-consensus cohort).** The table describes the composition of standardised PN formulations in the post-consensus cohort. Additional file 3:
**Major differences in PN practice between pre-consensus and post-consensus cohorts.** This table summarises the major improvements in the post-consensus cohort in comparison to pre-consensus cohort.

## Abbreviations

AAD: Amino acid dextrose
ANZ: Australia and New Zealand
ESPGHAN: European Society of Paediatric Gastroenterology, Hepatology and Nutrition
IQR: Interquartile range
NICU: Neonatal Intensive Care Unit
PMA: Postmenstrual age
PN: Parenteral nutrition
RHW: Royal hospital for women
SPN: Standardized parenteral nutrition
