# Supplementary material for: Improved nutrient intake following implementation of the consensus standardised parenteral nutrition formulations in preterm neonates – a before-after intervention study
Source: BMC Pediatr. 2014 Dec 17;14:309. doi: 10.1186/s12887-014-0309-0 (PMC4275977; doi:10.1186/s12887-014-0309-0)
Supplement: Additional file 1: — Standardised Amino acid-Dextrose Formulations from January 2010 to June 2011 (Pre-consensus cohort). The table describes the composition of standardised PN formulations in the pre-consensus cohort. [file 12887_2014_309_MOESM1_ESM.doc]

**Additional File 1**

Standardised Amino acid-Dextrose Formulations from January 2010 to June 2011 (Pre-consensus cohort)

|  | Preterm PN 1 | Preterm PN 2 | Preterm High Na | Preterm 7.5% Dextrose | Term PN |
| --- | --- | --- | --- | --- | --- |
| Indications | Starter PN.  Ideal solution to start within hours of birth. | Standard preterm PN solution.  Usually start after 48 hr | For hyponatremic preterm infants | For hyperglycaemic VLBW infants | Standard PN solution for term infants |
| Conc/Litre | | | | |  |
| Protein, g | 20 | 20 | 20 | 20 | 20 |
| Glucose, g | 100 | 100 | 100 | 75 | 100 |
| Na, mmol | 0 | 30 | 60 | 30 | 15 |
| K, mmol | 0 | 20 | 20 | 20 | 15 |
| Cl, mmol | 4 | 20 | 35 | 20 | 25 |
| Ca Gluconate, mmol | 12 | 12 | 12 | 12 | 9 |
| Mg, mmol | 2.5 | 2.5 | 2.5 | 2.5 | 2.5 |
| Ph, mmol | 0 | 9 | 9 | 9 | 9 |
| Zn, µg | 0 | 50 | 50 | 50 | 40 |
| Acetate, mmol | 0 | 32 | 40 | 32 | 0 |
| Bag volume | 750 ml | 750 ml | 750 ml | 750 ml | 1000 ml |
| At 150 ml/Kg/Day | | | | |  |
| Protein  g/kg/day | 3 | 3 | 3 | 3 | 3 |
| Na, mmol/kg/day | 0 | 4.5 | 9 | 4.5 | 2.25 |
| K, mmol/kg/day | 0 | 3 | 3 | 3 | 2.25 |
| Cl, mmol/kg/day | 0.6 | 3 | 5.25 | 3 | 3.75 |
| Acetate  Mmol/kg/day | 0 | 4.8 | 6 | 4.8 | 0 |
| Ca, mmol/kg/day | 1.8 | 1.8 | 1.8 | 1.8 | 1.35 |
| Ph, mmol/kg/day | 0 | 1.35 | 1.35 | 1.35 | 1.35 |
| Zinc, ug/kg/d | 0 | 7.5 | 7.5 | 7.5 | 60.6 |
| Mg, mmol/kg/day | 0.375 | 0.375 | 0.375 | 0.375 | 0.375 |
