# Supplementary material for: Improved nutrient intake following implementation of the consensus standardised parenteral nutrition formulations in preterm neonates – a before-after intervention study
Source: BMC Pediatr. 2014 Dec 17;14:309. doi: 10.1186/s12887-014-0309-0 (PMC4275977; doi:10.1186/s12887-014-0309-0)
Supplement: Additional file 2: — Standardised Amino acid-Dextrose formulations from July 2011 (post-consensus cohort). The table describes the composition of standardised PN formulations in the post-consensus cohort. [file 12887_2014_309_MOESM2_ESM.doc]

**Additional File 2**

|  | Starter PN | Standard Preterm PN | High Na Preterm PN | 7.5% D Preterm PN | Term PN |
| --- | --- | --- | --- | --- | --- |
| Indications | From birth (preterm and term) & upto 120 ml/kg/d | Usually start after 24-48 hr | Hyponatremic preterm infants | Infants with hyperglycaemia | Term infants from 24-48 hours |
| Conc/Litre | | | | |  |
| AA, g | 33 | 30 | 30 | 30 | 23 |
| Glucose, g | 100 | 100 | 100 | 75 | 120 |
| Na, mmol | 15 | 33 | 60 | 33 | 25 |
| K, mmol | 0 | 22 | 22 | 22 | 20 |
| Cl, mmol | 12 | 16 | 36 | 22 | 26 |
| Ca, mmol | 12 | 12 | 12 | 12 | 12 |
| Mg, mmol | 1.5 | 1.5 | 1.5 | 1.5 | 1.5 |
| Ph, mmol | 10 | 10 | 10 | 10 | 10 |
| Acetate, mmol | 5 | 40 | 44 | 40 | 13.5 |
| Zinc, µg | 0 | 3260 | 3260 | 3260 | 1900 |
| Selenium, µg | 0 | 20 | 20 | 20 | 20 |
| Iodine, µg | 0 | 8 | 8 | 8 | 8 |
| Heparin, units | 500 | 500 | 500 | 500 | 500 |
| Osmol, mosm/L | 813 | 790 | 790 | 651 | 847 |
| At 135 ml/Kg/Day | | | | |  |
| AA, g/kg/day | 4.5 | 4 | 4 | 4 | 3 |
| Glucose, g/kg/day | 13.5 | 13.5 | 13.5 | 10 | 16.2 |
| Na, mmol/kg/day | 2 | 4.5 | 8.1 | 4.5 | 3.4 |
| K, mmol/kg/day | 0 | 3 | 3 | 3 | 2.7 |
| Cl, mmol/kg/day | 1.6 | 2.2 | 4.9 | 3 | 3.5 |
| Acetate  mmol/kg/day | 0.7 | 5.4 | 6 | 5.4 | 1.8 |
| Ca, mmol/kg/day | 1.6 | 1.6 | 1.6 | 1.6 | 1.6 |
| Ph, mmol/kg/day | 1.4 | 1.4 | 1.4 | 1.4 | 1.4 |
| Mg, mmol/kg/day | 0.2 | 0.2 | 0.2 | 0.2 | 0.2 |
| Zinc, ug/kg/d | 0 | 440 | 440 | 440 | 256 |
| Selenium, ug/kg/d | 0 | 2.7 | 2.7 | 2.7 | 2.7 |
| Iodine, ug/kg/d | 0 | 1 | 1 | 1 | 1 |

Standardised Amino acid-Dextrose formulations from July 2011 (post-consensus cohort)
