# Supplementary material for: Improved nutrient intake following implementation of the consensus standardised parenteral nutrition formulations in preterm neonates – a before-after intervention study
Source: BMC Pediatr. 2014 Dec 17;14:309. doi: 10.1186/s12887-014-0309-0 (PMC4275977; doi:10.1186/s12887-014-0309-0)
Supplement: Additional file 3: — Major differences in PN practice between pre-consensus and post-consensus cohorts. This table summarises the major improvements in the post-consensus cohort in comparison to pre-consensus cohort. [file 12887_2014_309_MOESM3_ESM.doc]

**Additional File 3**

Major differences in PN practice between pre-consensus and post-consensus cohorts

| Parameters | Pre-Consensus | Post-Consensus |
| --- | --- | --- |
| PN Management Protocol | No | Yes |
| Protein intake | 20gm/L PN solution  Aim: 3g/kg | 33g/L &30g/L PN solution  Aim: To start 2g/kg and maximum 4g/kg |
| Lipid intake | 20% Clinoleic solution  Aim: Start 1g/kg and increase by 1g/kg each day to max 3g/kg  Monitoring: None | 20% Clinoleic solution  Aim: Start 1g/kg and increase by 1g/kg each day if triglyceride level is <2.8 mmol/L to max 3g/kg  Monitoring: Check triglyceride level before each increase, |
| Glucose | 10% &7.5% PN solution | 10% &7.5% PN solution |
| Sodium | 30 mmol/L  Aim:4.5 mmol/kg | 33 mmol/L  Aim:4.5 mmol/kg |
| Potassium | 20 mmol/L  Aim:3 mmol/kg | 22 mmol/L  Aim:3 mmol/kg |
| Chloride | 20 mmol/L  Aim:3 mmol/kg | 16 mmol/L  Aim:2.2 mmol/kg |
| Acetate | 32 mmol/L  Aim:4.8 mmol/kg | 40 mmol/L  Aim:5.4 mmol/kg |
| Calcium | 12 mmol/L  Aim:1.8 mmol/kg | 12 mmol/L  Aim:1.6 mmol/kg |
| Magnesium | 2.5 mmol/L  Aim:0.375 mmol/kg | 1.5 mmol/L  Aim: 0.2mmol/kg |
| Phosphate | 9 mmol/L  Aim:1.35mmol/kg | 10 mmol/L  Aim: 1.4mmol/kg |
| Zinc, µg | 50  Aim:7.5 µg/kg | 3260  Aim: 440µg /kg |
| Selenium, µg | - | 20  Aim: 2.7µg /kg |
| Iodine, µg | - | 8  Aim: 1µg /kg |
| Maximum Fluid intake | 150ml/kg: AA-Dex solution  Lipid: Extra | 150ml/kg: AA-Dex solution  Lipid: Included in total |
